# Different Innovative Laser Implants Characteristics Histomorphometric and SEM-EDX Comparison for In Vivo Applications

**DOI:** 10.3390/ma18133165

**Published:** 2025-07-03

**Authors:** Filiberto Mastrangelo, Marco Cicciù, Raimondo Quaresima, Antonio Scarano

**Affiliations:** 1 Department of Clinical and Experimental Medicine, University of Foggia, Via Rovelli n. 60, 77100 Foggia, Italy; 2 Department of Biomedical and Surgical and Biomedical Sciences, Catania University, 95123 Catania, Italy; mcicciu@unime.it; 3 DICEAA, Department of Civil, Construction-Architectural and Environmental Engineering, University of L’Aquila, 67100 L’Aquila, Italy; raimondo.quaresima@univaq.it; 4 Department of Innovative Technologies in Medicine & Dentistry, University of Chieti-Pescara, 66013 Chieti, Italy; ascarano@unich.it

**Keywords:** osseointegration, laser titanium implants, sandblasted and acid-etched implants, SEM-EDX analysis, bone-to-implant contact, dynamic osseointegration index, bone quality index, wettability

## Abstract

Objectives: In the animal model, we aim to evaluate the bone behavior in two innovative and different laser-treated (L1–L2) titanium implants compared to sandblasted and acid-etched (SBAE) used as control. Materials and Methods: A total of twenty-seven dental implants (8.5 × 3.3 mm) used for the study (Sweden & Martina, Due Carraie Padova-Italy) were placed in three Pelibuey female sheep. Implant surface profilometric, contact angle and EDX analysis were detected. After 15, 30 and 90 days, histological, histomorphometric, SEM-EDX analysis and Bone-to-implant Contact (BIC), Dynamic Osseointegration Index (DOI) and Bone Quality Index (BQI) (as Calcium and Phosphorous atomic percentages ratio) were performed. Results: All surfaces showed relevant profilometric and wettability differences. After 15 days, BIC_15_ showed great differences in L2 (42.1 ± 2.6) compared to L1 (5.2 ± 3.1) and SBAE (23.3 ± 3.9) as well as after 30 days (L2 (82.4 ± 2.2), L1 (56.2 ± 1.3) and SBAE (77.3 ± 0.4)). After 90 days, relevant lower BIC_90_ values were detected in L1 (68.4 ± 0.2) compared to L2 (86.4 ± 0.1) and SBAE (86.2 ± 0.6). The DOI showed higher rates of bone growth in L2 after 15 (DOI_15_ = 2.81) and 30 days (DOI_30_ = 2.83), compared to L1 (DOI_15_ = 0.38, DOI_30_ = 3.40) and SBAE (DOI_15_ = 1.55, DOI_30_ = 2.58). The DOI_90_ drastic slowdown in SBAE (0.96), L1 (0.76), and L2 (0.95) confirmed the Early Osseointegration (EO) as a crucial phase. Moreover, before loading, the lower global BQI in L1 (Ca 44.43 ± 0.08–P 46.14 ± 5.15) and SBAE (Ca 45.31 ± 2.08–P 48.28 ± 1.12) compared to L2 (Ca 79.81 ± 2.08–P 81.85 ± 3.14) allows to assert that osseointegration process and bone healing could not be considered complete if compared to the native bone. Conclusions: The BIC, DOI, and BQI results showed that osseointegration is a dynamic process, confirming the crucial role of surface characteristics able to influence it, especially the early osseointegration (EO) phase. The short-time L2 implants’ higher bone quantity and quality results, compared to L1 and SBAE, suggested the fundamental role of this innovative laser-obtained surface in “secondary stability” and predictable long-term clinical outcomes.

## 1. Background

Dental implants that replace missing teeth and rehabilitate completely or partially edentulous patients have become a predictable option with excellent long-term clinical results [1,2].

During the last sixty years, several studies were promoted to improve the knowledge around the “osseointegration” concept of the commercially pure titanium implant, based on Branemark’s definition as “a direct contact—on light microscopic level—contact between living bone and implant” [3,4,5].

During the 1980s–1990s, several technologies and titanium surface treatments were tested to improve the bone apposition around titanium screws [6,7] and promote osseointegration [8,9,10,11], establishing that the moderate roughness (or micro-roughness) obtained by large grit sandblasting and acid etching technology, promoted significantly increased removal torque value, defined by Buser as “the first choice for clinical application” [12].

This statement produced more than a thousand different implant systems in vitro and in vivo testing, with similar macroscopical (shape, dimension, design, color and connection) and microscopical (surface roughness topography, wettability, composition, and coating) [13,14] characteristics.

Although there are high long-term success rates of dental implants, the presence of implant failure and aesthetic failure in several cases, together with the increase of patients’ requests to reduce the prosthetic rehabilitation time and improve the immediate loading, nowadays raises the question of there is a new technology or approach, capable of accelerating the osseointegration and clarifying the several doubts that still exist around the biological process of ankylosis of the titanium implants into the bone [2,15,16,17,18,19].

The bone-to-implant contact (BIC) technique, obtained with optical microscopy histomorphometric analyses, showed only the static quantitative bone response around the fixture. In any case, the necessity to evaluate the bone quality and describe the dynamic bone behavior during osseointegration remains a clinical and scientific priority [20,21].

Recently, Dynamic Osseointegration Index (DOI) and Bone Quality Index (BQI) were proposed to assess the bone dynamic growth and the mineralization rate of the hard tissue during healing time around titanium [2,15].

The laser use for implant surface treatment is not new [22,23], but it still shows relevant advantages compared to traditional manufacturing methods, promoting well control and reproducible surface texture (roughness and waviness) and reducing the production time. Also, the absence of surface contaminants, the surface cleaning and sterilization of the fixture system, together with an environmental and manipulation reduction impact, seem an important evolution if supported by biological results when compared to the traditional implants [16].

The aim of the present pre-clinical animal study was to assess the following:

1. The early and late osseointegration process around two different innovative laser implants (L1–L2) compared to sandblasted and acid-etched (SBAE) used as controls;
2. If laser treatment and the chemical and physical surface properties influenced bone healing;
3. If the laser technology is able to improve the osseointegration compared to sandblasted and acid-etched (SBAE) used as controls.
4. The quality and the bone behavior during the osseointegration process.

## 2. Materials and Methods

### 2.1. Implant, SEM, Energy Dispersive X-Ray Spectroscopy (EDX) and Profilometric Analysis

To prevent errors or bias, and according to the sheep tibia dimension, a total of twenty-seven dental implants with Commercially pure (Cp-Ti) Ti screws (Sweden & Martina, Due Carraie, Padova, Italy) 3.3 mm diameter and 8.5 mm height were used. Nine dental implant surfaces were obtained with a zirconia sandblasting and acid etching (SBAE) treatment, while eighteen implants were Ytterbium laser-treated to obtain two distinct surfaces (L1–L2) with specific different profilometric characteristics. All specimens were analyzed with Scanning Electron Microscopy (SEM) (Zeiss GeminiSEM500, Jena, Germany) and laser profilometer (NP-Flex-1000, Bruker, Randwick, Australia) to obtain Profile heights (Ra), Root mean square average (Rq), Profile average maximum height (Rz), maximum height Profile (Ry), and Mean Spacing between peaks (Sm). Roughness analyses, according to ISO 21920-2:2021, were performed in triplicate for each fixture along linear measurements of 8 mm (points density of 500/mm) of the external fixture profile with a cut-off of 0.25, 0.80, and 0.10 mm, 2RC and phase correct Gaussian filters, and a transmission of the selected cut-off to 75% as reported by the manufacture specification. The *t*-test using Holm-Sidak software (version 2.0.0.0) was used to evaluate the statistical significance (*p* < 0.01 (*), *p* < 0.05 (**) and *p* ≤ 0.005 (***)) of the normal distribution of the surface roughness parameter values. The *p* ≤ 0.005 between the samples was considered statistically significant. Concerning implant, chemical surface analysis was assessed with Energy Dispersive X-ray Spectroscopy (EDX) 20 kV for 1 min (OXFORD EDS Oxford Aztec Live Ultim Max 100, Abingdon, UK).

### 2.2. Wettability

Surface static contact angle (CA) was recorded to evaluate the surface wettability and hydrophilic-hydrophobic behavior using a contact angle analysis (GBX Scientific Instruments, Romans, France).

A total of nine disks were made and shared in 3 groups. Three flat surfaces, respectively two lasers (L1–L2) and one sandblasted and acid-etched (SBAE) treated, were specifically prepared. On each surface, a distilled water drop (2.0 μL) was placed in three different spot areas, and the CA was recorded within 1 s. Nine measurements were obtained from each type of surface tested, and the mean value and standard deviation were used for the CA analysis.

### 2.3. Surgical Protocol

The study protocol “A new laser treated implant surface: experimental pilot study in the tibia of the sheep” was conducted according to the guidelines of the Declaration of Helsinki and approved by (number 05/2018) by the Ethics Committee of the University of La Havana (Cuba) where the surgical procedures were performed. The ARRIVE list of surgical procedures and care (Essential Information and Recommended Set) was strictly followed. Three Pelibuey female sheep in good general health were selected. To reduce the odds and to reproduce the natural bone healing conditions, the authors placed dental implants with the same height and diameter in sheep tibia. To minimize the potential errors and distinguish the 3 different implants, the collar surface was marked. At 15, 30 and 90 days, in each sheep tibia cortical bony crest, 3 different implants (L1, L2 and SBAE) were placed. After 90 days, the animals were first anesthetized and then euthanized. All specimens were retrieved into bone block sections and preserved in 10% buffered formalin.

### 2.4. Histomorphometric Analysis and Bone-to-Implant Contact (BIC) Percentage Evaluation

Dehydrated (graded ethanol series) and embedded with glycol methacrylate resin (Technovit 7200 VLC Kulzer, Friedrichsdorf, Germany) specimens, transversally cut along the implant centreline (Exakt1, Apparatebau, Norderstedt, Germany-ATM QNESS-GMBH, Mammelzen, Germany) (60 μm slices thickness) were ground (silicon carbide P280, P320, P400, P600-ISO/FEPA Grit designation) and diamond-polished (polycrystalline 9.3 and 1 μm suspension) to obtain a thickness around 15–20 μm.

The histological sections were Stevenel’s blue and Alizarin Red S stained analyzed by transmitted microscope (ZeissAxio Imager M2, Jena, Germany), recorded by a digital camera (Zeiss AxioCam 503 MONO1, Jena, Germany), and evaluated with digital pad (Wacom STU 540, Wacom Technology Corporation, Portland, OR, USA). The bone-to-implant Contact (BIC) percentage was performed at the University of Chieti-Pescara with histomorphometric software (Image-Pro Plus 4.5, Media Cybernetics Inc., Immagini & Computer Snc, Milano, Italy) as a ratio between the implants linear surface in contact with the mineralized bone, when compared to the total implant fixture profile.

### 2.5. Dynamic Osseointegration Index (DOI)

In order to assess the osseointegration bone apposition rate around the implant surface during all healing times, Dynamic Osseointegration Index (DOI) was detected as a ratio between the means of the BIC percentage mean value of the three different specimen groups and the numbers of experimental days (15, 30, and 90 days) [2,15].

### 2.6. Bone Quality Index (BQI)

**Figure 1:**
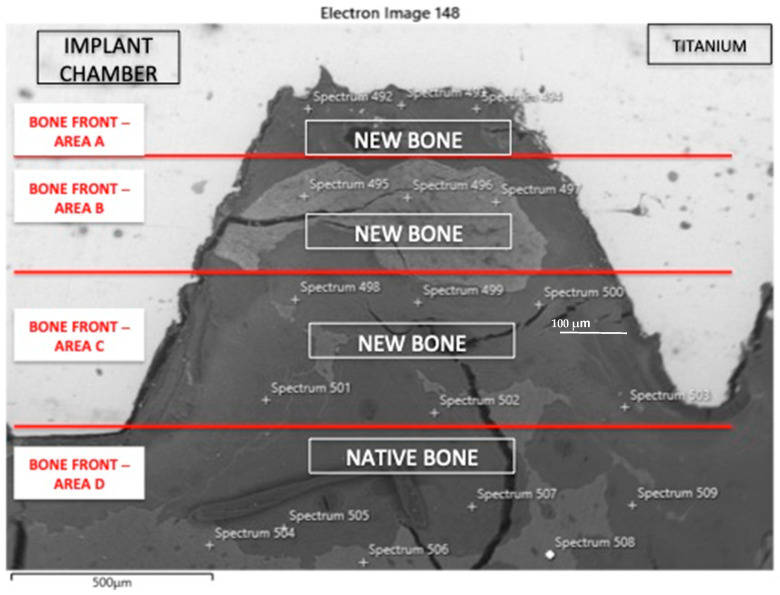
Scheme of the SEM-EDX analysis of the implant chambers 1, 3, and 5 to assess the BQI as a ratio between the Calcium (Ca) and Phosphorous (P) content in specific and reproducible areas. Bone Front in Area A: Interface Titanium-Bone Front; Bone Front in Area B: Middle Chamber Bone Front; Bone Front in Area C: Interface Native Bone—Surgical Treating area and Bone Front in Area D: native bone.

According to the previous scientific literature [24], the BQI value was able to provide some information about bone quality and mineralization around the dental implant. Since the bone is made up of hydroxyapatite and this showed a chemical formula Ca_10_(PO_4_)_6_(OH)_2_, the authors used the BQI as a ratio between the Calcium (Ca) and Phosphorous (P) contents obtained by SEM-EDX analysis (OXFORD EDS Oxford Aztec Live Ultim Max 100) to evaluate bone mineralization and maturation gradient through specific and reproducible 48 measurements in distinct Area of Interest (AOI) between two coils of the implant screw defined Implant Chambers 1-3-5 (Figure 1). After the surgical bone site preparation, this Implant Chamber space was initially empty, and after the implant placement into the bone, the space was filled by the clot and bone debris. During the osseointegration, the empty space will be filled by the new bone, allowing the ankylosis of the implant screw. Before implant loading, the BQI could be able to show some information about the bony front during healing times, the bone behavior, mineralization and maturation. In all specimens, the bony front was evaluated in three distinct implant chambers: (1) neck, (2) body and (3) apical areas of the screw. In each Chamber, 4 distinct areas were detected: Area A: Interface Titanium-Bone; Area B: Middle Chamber Bony Front; Area C: Interface Native Bone—Surgical Bone Treating; and Area D, representative of the native bone. In each chamber, a total of 48 EDX measurements and the T-student statistical analysis were performed. ZAF (number effect (Z) absorption (A), and fluorescence (F) correction methods were performed for normalized qualitative and semi-quantitative (weight % and atomic %) Ca and P content [2] (Figure 1). It is possible to achieve greater reproducibility with more and different measures [2,25,26,27,28,29], but must be taken into account more than the costs of the ethical and licensing issues. In any case, local bone variations due to remnants or debris can be taken into account, considering the standard deviations on Ca and P measures.

## 3. Results

All specimens placed into the sheep tibia showed no inflammation or necrosis area. No implant failure was observed. Additionally, all specimens showed osseointegration during histological, histomorphometric, and SEM analyses, although with main differences between the groups.

### 3.1. Electron Scanning Microscope (SEM) Analysis of Implants

**Figure 2:**
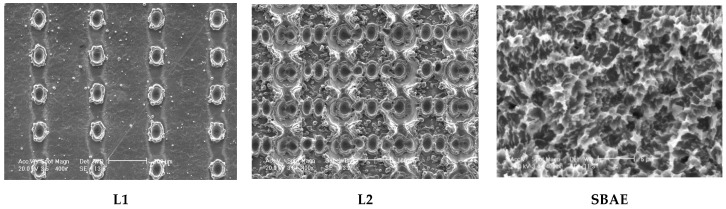
Scanning Electron Microscope (SEM) analysis at 400× of L1, L2 (400×) and SBAE (4000×) titanium surfaces (Scale bars: L1 and L2 100 µm, SBAE 5 µm).

**Figure 3:**
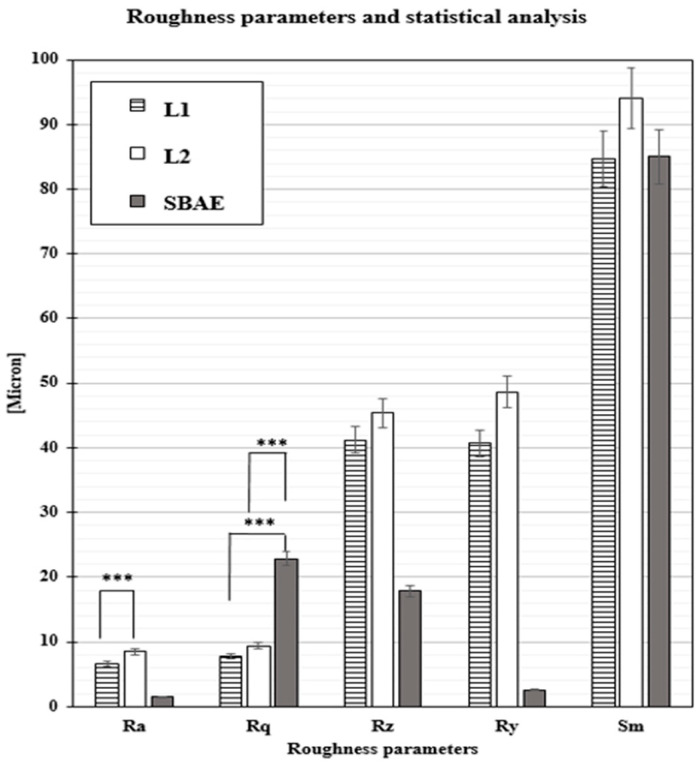
Profilometric evaluations of L1, L2, and SBAE titanium surfaces for roughness parameters (Ra, Rq, Rz, Ry, and Sm). (*** *p* ≤ 0.005).

The Scanning Electron Microscope (SEM) analysis of L1, L2, and SBAE showed significantly different macro and microscopic surface characteristics (Figure 2). In L1 surfaces, parallel lines of laser dots (50 µm in diameter) separated by smooth titanium were observed. The regular roughness was confirmed by the profilometric analysis (Figure 3). The L2 specimens showed extremely complex structures of several different dots. The complex surface micro-topography showed several round spikes of different heights alternated to wide and narrow valleys with different depths, as confirmed by the profilometric results (Figure 3). In SBAE specimens, a typical sandblasted and acid-etched flat structure was observed, consisting of several irregular and rounded peaks alternated by low valleys, as confirmed by the profilometric analysis (Figure 3).

### 3.2. Profilometric Analysis of Implants

The profilometric analysis showed different surface roughness characteristics for Ra, Rq, Rz, Ry, and Sm (Figure 3). The linear surface roughness (Ra) detected in the L2 samples (8.51 ± 0.84 µm) had a higher average value compared to those for L1 (6.58 ± 0.72 µm) and SBAE 1.51 µm (±0.18). The L1 (7.87 ± 0.91 µm) root-mean-square (Rq) evaluation showed a lower value compared to L2 (9.42 ± 0.94 µm) and SBAE (22.87 ± 1.30 µm). The average distance between the highest peak and lowest valley (Rz) was higher in L2 (45.35 ± 9.35 µm) than L1 (41.16 ± 8.25 µm) and SBAE (17.90 ± 1.37 µm), signaling a more complex and irregular surface texture. The maximum height of the Laser treatment surface profile (Ry) showed a similar value of 48.60 µm (±9.92) in L2 and 40.73 µm (±9.92) in L1, compared to the lower value of 02.62 µm (±0.12) in SBAE specimens. The mean spacing between peaks (Sm) showed similar values for L1 (84.70 ± 7.92 µm) and SBAE (85.00 ± 8.63 µm), compared to the higher value of L2 (96.33 ± 10.69 µm). The *t*-test relationship between the profilometric data recorded in L1 vs. L2 for Ra value (0.0013), in L1 vs. SBAE for Rq (0.0001), and in L1 vs. SBAE for Rq (0.0001), showed statistically significant differences (*** *p* ≤ 0.005) and was considered positive, confirming the extreme differences between the three groups of dental implant surfaces (Table 1).

### 3.3. Surface Chemical Characteristics and Wettability

Energy Dispersive X-ray Spectroscopy (EDX) chemical surface evaluation assessed only Titanium (Ti) and Oxygen (O) due to the Commercially Pure Titanium (Cp-Ti) implant composition. Similar hydrophilic wettability characteristics were shown by contact angle analysis of L1 (69.5 ± 3.2) and L2 (71.5 ± 2.4) surfaces, while in SBAE, different hydrophobic screw values were assessed (137.3 ± 2.6).

### 3.4. Histological and Histomorphometric Analysis

**Figure 4:**
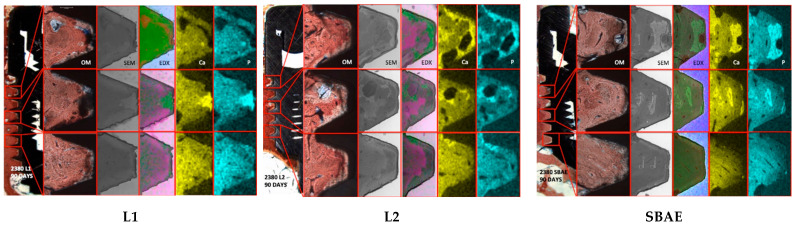
After 90 days, L1–L2–SBAE specimen chambers 1-3-5 were evaluated with an Optical Microscope (OM), Scanning Electron Microscope (SEM), Energy Dispersive X-ray (EDX) analysis, and Calcium (Ca) and Phosphorus (P) distribution Spectroscopy analysis during implant osseointegration.

Histological evaluation showed dynamic and discontinuous bone behavior in all specimens. After 15 days, several areas of the implant surface showed no bone contact. In the apical Area of all screws, only a few spots of bone were observed in contact with the implant surface. In L1, several bone gaps in the screw body were observed, compared to L2 and SBAE. After 30 days, a homogeneous bone layer completely covered the L2 implant surface, compared to the SBAE implants in which several immature bone areas were observed. In L1, some areas with completely absent bone connection to the implant surface were shown, especially around the apical implant area. After 90 days, the histological analysis showed the bone completely covered the implant surface in all specimens. After 90 days, only in L2 samples, typical mature bone with osteon architecture was observed in the neck and body areas of the screw (Figure 4).

The histomorphometric evaluation showed different bone behavior around the three groups of dental implants during the osseointegration healing period observed. A higher statistically significant BIC percentage was detected in L2 samples compared to SBAE and L1 (*p* < 0.05) in all phases of osseointegration. After 15 days, a higher BIC percentage (42.1 ± 2.6) was detected in L2 compared to L1 (5.2 ± 3.1) and SBAE (23.3 ± 3.9). After 30 days, a higher BIC value was observed in L2 (82.4 ± 2.2) compared to a lower value in L1 (56.2 ± 1.3), while SBAE specimens showed an important improvement in bone-to-implant contact (77.3 ± 0.4). After 90 days, very similar values were observed in L2 (86.4 ± 0.1) and SBAE (86.2 ± 0.6) specimens compared to a lower BIC value in L1 (68.4 ± 0.2) (Table 2).

### 3.5. Dynamic Osseointegration Index (DOI)

**Figure 5:**
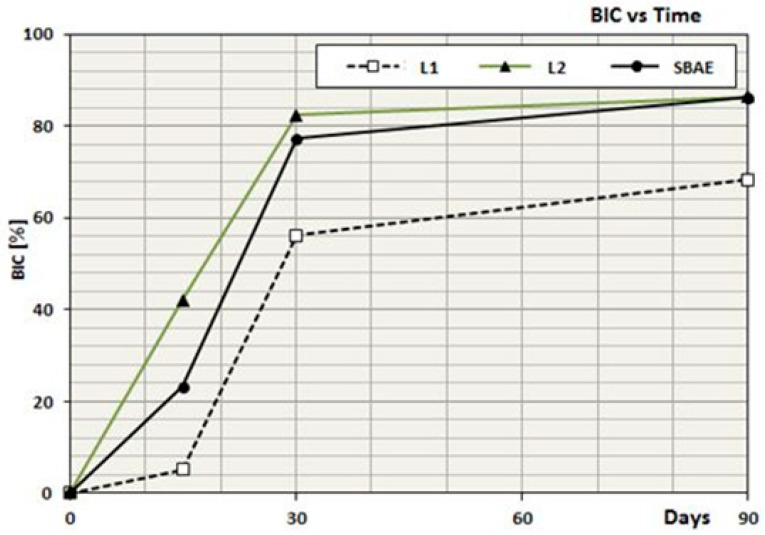
DOI Index: the BIC trend evolution over time.

During the healing time, very different bone behavior was observed in the three groups, with dynamic and non-constant improvement of the osseointegration. After 15 days, DOI showed a higher medium value in L2 (2.81) compared to SBAE (1.55) and L1 (0.38). After 30 days, greater improvement in L2 (3.40) was detected if compared to SBAE (2.58) and L1 (2.83), in which the medium values continued to be high. During this phase, only in L1 specimens does the bone apposition completely reach the titanium surface. After 90 days, a drastic reduction of bone apposition onto the titanium surface was detected with similar values in L2 (0.95) and SBAE (0.96), compared to L1 (0.76) (Table 2) (Figure 5).

### 3.6. Bone Quality Index (BQI)

At the Interface, Titanium-Bone Front (Area A) Ca and P BQI mean values showed different bone mineralization in all samples. After 15 days, Ca (31.96 ± 02.91) and P (33.97 ± 02.98) levels were higher in L2 specimens, compared to SBAE (Ca:25.35 ± 02.01/P: 27.16 ± 03.15) and L1 (Ca: 19.49 ± 02.08/22.45 ± 01.78), which were significatively lower. After 30 days, the specimens showed very similar low BQI mean values in the same area in L1 (Ca: 24.00 ± 05.55/28.50 ± 06.35) and SBAE (Ca: 25.91 ± 01.90/26.86 ± 03.15), compared to L2 (31.23 ± 07.18/32.84 ± 07.74). After 90 days, the SEM-EDX analysis showed different Ca and P ion values with further improvement in L2 (35.81 ± 04.22/38.85 ± 02.75), compared to L1 (25.57 ± 05.24/27.03 ± 08.16) and SBAE (26.25 ± 06.27/27.82 ± 06.49) (Table 3).

In all specimens, the BQI showed different bone mean values in the A, B, and C Areas of the 1, 3, and 5 implant chambers evaluated. After 15 days, there was a significant high BQI mean value in L2 (Ca:35.15 ± 1.80/P37.76 ± 1.87) compared to SBAE (Ca:22.99 ± 1.00/P: 23.21 ± 1.01) and L1 (Ca: 20.59 ± 1.08/21.55 ± 1.48) and after 30 days, the greatest BQI improvement in L2 (63.94 ± 0.80/71.84 ± 3.21), compared to 15-day previously marked, and if compared to L1 (Ca:39.54 ± 2.38/39.09 ± 3.29) and SBAE (Ca:39.23 ± 2.00/43.45 ± 4.58) specimens. After 90 days, further improvements were seen in L2 (79.81 ± 2.08/81.85 ± 3.14), compared to L1 (44.43 ± 0.08/46.14 ± 5.15) and SBAE (45.31 ± 2.08/48.28 ± 1.12), where BQI showed moderate increases due to similar values (Table 4).

## 4. Discussion

Currently, dental implantology is considered a standard clinical practice with a high predictable success rate [2,30,31,32,33,34] to achieve stable and long-lasting results [2,4,5,6,7,16,35,36]. Due to the growing demands to replace lost teeth, several studies have been mainly oriented on clinical aspects, such as reduction of surgical trauma, primary implant stability and aesthetic improvement, healing time reduction, minimally invasive procedures and immediate loading promotion [36,37,38,39,40].

### 4.1. Histological Analysis

The no implant failure and the histologically relevant vital bone surrounded all the implants with the absence of inflammation, reabsorption or necrosis areas, and the global new bone formation around all specimens confirmed the biocompatibility of all dental implants and the technological procedures to obtain it. The extremely variable wettability results in L2 (hydrophilic) and SBAE (hydrophobic), when correlated with the BIC recorded, seem not able to influence lonely osseointegration. On the contrary, the higher Ra (profile heights) profilometric values in correlation with the lower Rq (root mean square) values recorded in L2 implants BIC-BQI-DOI results, compared to SBAE and L1 implants, seem to play an active role in faster osseointegration and crucial especially in the early phase (EO). The results seem to confirm that the complex surface microstructure in L2, compared to a regular and/or low surface structure in L1 or SBAE, is crucial for the osseointegration and not the technology with which the surface microstructure is obtained.

### 4.2. Histomorphometric Analysis

In the last decades, several studies have been promoted to evaluate whether the surface characteristics are able to influence the BIC. However, many questions are still open. Indeed, in 2008, Yeo [40] showed how the design factor seems not to play a role in the early bone response, as well as, in 2012, Choi [41] and in 2024 Kunrath [42] in different surface-modified titanium implants, detected no significant difference in the bone response two weeks after implant placement. On the contrary, in 2009, Lang [43] and 2010 Wennerberg [17] affirmed that the surface properties seem to determine the osseointegration. Moreover, the SLA implant BIC values are also more different, as showed in a dog model (40.91%) by Koch after 4 months [44] or by Thoma after 6 months (87.85%) [45], in a minipigs model (69.3%) by Gahlert after 2 months [46] or by Schliephak after 3 months (79.80%) [47] or again by Gahlert after 4 months (58.5%) [48], as well as showed in rabbits by Aboushlelib after 1 month (56.9%) [49] and in rat by Kohal after 9 months (72.9%) [50].

Also, in 2000, Ricci [51] and Weiner in 2008 [52] showed a closer bone induced by laser micro-textured surfaces in the BioLok Laser-Lok^®^ Implant system. As well as in 2016, in a sheep animal model, Trisi [53] showed a significantly higher BIC percentage in laser-treated implants compared to machined implant surfaces.

The histomorphometric analysis of the present study showed higher BIC value compared to the current scientific literature in the sheep animal model [20,36,37,38,39] and several intra-group implant BIC value differences. In the same animal model, the high BIC values recorded in L2 after 15 (42.1) and 30 days (82.4) compared to the lower SBAE and L1 values at the same time, confirmed that the implant surface is capable of directly influencing the bone-to-implant contact in less time than others. After 90 days, the BIC slow recovery (86.2) in SBAE implants compared to L1 (86.4) and the L2 very low value (68.4) recorded confirmed the surface crucial role in early and late osseointegration. Also, L2 BIC study results are still higher if compared to BIC reported in machined, acid, sandblasted, and zirconia implant surfaces reported by Hafezeqoran [54] and De Tullio in a recent systematic review and meta-analysis [55,56]. After all, the BIC study results show that not all implant surfaces should be recommended by clinicians for immediate or early implant placement and used to reduce the risk of rehabilitation failure in dental implants’ immediate loading procedures.

### 4.3. Bone Behavior During Osseointegration

In addition to the bone-to-implant contact percentage (BIC), to better understand the dynamic osseointegration process and the bone behavior, the Dynamic Osseointegration Index (DOI) quantifies the bone growth and speed, and the Bone Quality Index (BQI) evaluates the bone quality and mineralization around implants.

After 15 days, L2 DOI value showed rapid and fast bone growth, double if compared to SBAE and more than triple if compared to L1. This trend increased constantly until 30 days, showing the dynamic bone behavior with different speeds between the specimens. Until 90 days, the decreased speed observed in L2 compared to SBAE and L1 is a normal phenomenon related to the complete bone anchorage to the implant surface.

DOI trend showed very well the dynamic bone behavior and the crucial role played by high Ra, low Rq value and low hydrophobicity, especially in the EO osseointegration [2,15]. The BIC and DOI improvement could be related to the different capabilities of the blood clot to adhere to the L2 complex surface microtopography compared to smooth SBAE–L1 [20,35,41,56].

The bone atomic percentage of Calcium (Ca) and Phosporous (P) with SEM–EDX analysis and their ratio in Bone Quality Index (BQI) evaluated the quality and mineralization of the new bone grown around the dental implant (Areas A-B-C) during the osseointegration and compared to the native bone (Area D) [15]. The BQI results have shown how the bone mineralization proceeds in two distinct directions around the implants, vertical or “contact bone growth” onto the implant surface and horizontal or “at distance”, from the native bone to the implant screw.

The significant difference between the implant’s BQI results showed constant double values in L2 after 15–30–90 days, compared to SBAE and L1. The results confirmed the crucial role of the implant surface in influencing the new bone quality and mineralization.

During EO, the BQI values in B (50%) and C (75%) Areas, respectively, confirmed progressively higher degrees of bone mineralization and maturation in the horizontal direction.

After 90 days, in the same areas, 90% of BQI values were detected, confirming that the bone maturation in late osseointegration (LO) undergoes an acceleration.

Furthermore, the L2 BQI results in Area A (Bony Front—Titanium Surface) showed a stable higher mean value compared to SBAE and L1.

All BQI results have confirmed that bone quality and mineralization do not undergo changes during bone growth, it is influenced by the micro-shape implant surface, that the difference in quality begins in (EO) early osseointegration and that it continues in a constant and stable manner during bone apposition and growth, and finally, that the bone behavior cannot be evaluated only with the BIC scale, which instead shows a decrease in time and speed during late osseointegration (LO).

In conclusion, even though this study is limited by the number of animals used and implant grafted after 90 days, the BQI results in Area A have shown a low Ca/P ratio compared to the native bone, suggesting that in all specimens, the bone around the implants before loading, is not yet considered completely mature [2,15,57,58,59,60,61,62,63,64].

## 5. Conclusions

Further studies with a larger number of implants, with a longer observation period, and with and without prosthetic loading will be needed to confirm the interesting results obtained to develop efficient technologies and new standards manufacturers for a new generation of dental implants in relationship to the recent requests and higher quality standard of the patients.

The results of the present study confirmed the biocompatibility of the innovative dental implant laser obtained (L2) and a more rapid and predictable quality and quantity of the bone anchorage compared to SBAE, confirming this traditional technology does not seem to be now the first choice for clinical application.

Furthermore, the results detected that osseointegration around the implants is dynamic and not a constant process.

The results confirmed the active role of the surface complex characteristics (correlation between high hydrophilic, high Ra and lower Rq profilometric value) able to influence the speed, quality and quantity of bone growth, especially in clinical post-extractive or immediate loading rehabilitation.

The high BIC rate shown in all specimens compared to the literature seem to suggest that all implants were considered able for the implants rehabilitation.

Nevertheless, if the results were analyzed deeply, several differences were observed between the implant groups, confirming that nowadays, the BIC alone does not explain the biological process and the clinical implications of replying to the patient’s requests if not related to another osseointegration index as DOI and BQI.

In conclusion, with the limitations of the pilot study, the BQI results before implant loading confirmed the very poor bone mineralization after 90 days, and bone healing cannot be considered complete.

## Figures and Tables

**Table 1:** The Roughness parameters (Ra, Rq, Rz, Ry, and Sm) and statistically significant *t*-test relationship between profilometric data of the three dental implant groups, L1, L2, and SBAE. The *p*-value ≤ 0.005 was considered positive. Only Ra of L1 vs. L2 (0.0012), Rq of L1 vs. SBAE (0.0001), and 1 vs. SBAE (0.0001) showed statistically significant differences in surface textures (* *p* < 0.01, ** *p* < 0.05, *** *p* ≤ 0.005).

Roughness Parameters *t*-Test (*p*-Value)	Ra	Rq	Rz	Ry	Sm
L1 vs. L2	0.00123 ***	0.0549 *	0.2966 *	0.1935 *	0.1053 *
L1 vs. SBAE	0.0395 **	0.0001 ***	0.0172 **	0.0077 **	0.1148 *
L2 vs. SBAE	0.0201 **	0.0001 ***	0.0143 **	0.0107 **	0.4837 *

**Table 2:** Comparison in Area A of BIC, DOI and BQI results in L1, L2 and SBAE specimens after 15, 30, and 90 days.

Specimens		15 Days Area A	30 Days Area A	90 Days Area A
L1	BIC	5.2	56.2	68.4
	DOI	0.38	3.40	0.76
	Ca	19.49	24.00	25.57
	P	22.45	28.50	27.03
L2	BIC	42.1	82.4	86.4
	DOI	2.81	2.83	0.95
	Ca	31.96	31.23	35.81
	P	33.97	32.84	38.85
SBAE	BIC	23.3	77.3	86.2
	DOI	1.55	2.58	0.96
	Ca	25.35	25.9	26.25
	P	27.16	26.86	27.82

**Table 3:** The BQI mean value at the Interface between Bony Front—Titanium Surface (Area A) in L1, L2 and SBAE specimens after 15, 30, and 90 days. The interface between the Native Bone—Surgical Treating area represents Area D to compare the BQI value.

BQI (%) Mean Value	15 Days	30 Days	90 Days
	Area A		
L1			
Ca	19.49 ± 02.08	24.00 ± 05.55	25.57 ± 05.24
P	22.45 ±01.78	28.50 ± 06.35	27.03 ± 08.16
L2			
Ca	31.96 ± 02.91	31.23 ±07.18	35.81 ± 04.22
P	33.97 ± 02.98	32.84 ±07.74	38.85 ± 02.75
SBAE			
Ca	25.35 ± 02.01	25.91 ± 01.90	26.25 ± 06.27
P	27.16 ± 03.15	26.86 ±03.15	27.82 ± 06.49
L1–L2–SBAE	Area D		
Ca	100.00 ± 0.00	100.00 ± 0.00	100.00 ± 0.00
P	100.00 ± 0.00	100.00 ± 0.00	100.00 ± 0.00

**Table 4:** L1, L2 and SBAE BQI mean values in Areas A-B-C of the Chambers 1-3-5 after 15, 30, and 90 days. The Ca and P values in Area D represent the mineralization gradient of the native bone used as a reference for other compared areas.

Specimens	BQI_15_ Mean Value	BQI_30_ Mean Value	BQI_90_ Mean Value
L1	Areas A-B-C		
Ca	19.49 ± 2.08	39.54 ± 2.38	44.43 ± 0.08
P	22.45 ± 1.78	39.09 ± 3.29	46.14 ± 5.15
L2	Areas A-B-C		
Ca	35.15 ± 1.80	63.94 ± 0.80	79.81 ± 2.08
P	37.76 ± 1.87	71.84 ± 3.21	81.85 ± 3.14
SBAE	Areas A-B-C		
Ca	22.99 ± 1.00	39.23 ± 2.00	45.31 ± 2.08
P	23.21 ± 1.01	43.45 ± 4.58	48.28 ± 1.12
L1-L2-SBAE	Area D	Area D	Area D
Ca	100.00 ± 0.00	100.00 ± 0.00	100.00 ± 0.00
P	100.00 ± 0.00	100.00 ± 0.00	100.00 ± 0.00

## Data Availability

The original contributions presented in the study are included in the article, further inquiries can be directed to the corresponding author.
